# Current non-pharmacological practices for cancer-related fatigue in oncology rehabilitation: results of a national provider survey

**DOI:** 10.1007/s00520-026-10717-8

**Published:** 2026-05-08

**Authors:** Shana E. Harrington, Jeannette Q. Lee, Anne B. Fleischer, Kelley C. Wood, Joy C. Cohn, Daniel J. Malone, Mary Insana Fisher

**Affiliations:** 1 Department of Exercise Science, DPT Program, University of South Carolina, Columbia, SC USA; 2 UCSF/SFSU Graduate Program in Physical Therapy, San Francisco State University, San Francisco, CA USA; 3 Occupational Therapy Program, Department of Rehabilitation, Exercise and Nutrition Sciences, College of Allied Health, University of Cincinnati, Cincinnati, OH USA; 4 ReVital Cancer Rehabilitation, Select Medical, Mechanicsburg, PA USA; 5 Good Shepherd Penn Partners Rehabilitation, Philadelphia, PA USA; 6 Physical Therapy Program, University of Colorado Anschutz Medical Campus, Denver, CO USA; 7 Department of Physical Therapy, University of Dayton, Dayton, OH USA

**Keywords:** Neoplasm, Cancer, Survivorship, Treatment, Intervention

## Abstract

**Purpose:**

Cancer-related fatigue (CRF) is one of the most common side effects of cancer treatment, yet there is little consensus on best non-pharmacologic practices to manage this debilitating side effect. The purpose of this study was to describe current non-pharmacologic practice patterns for managing CRF and barriers to rehabilitation.

**Methods:**

An electronic survey, developed by the authors using the CHERRIES checklist, was piloted via REDCap™ by nine participants. Data collected included demographics, clinical experience, frequency (none of the time, some of the time, and all of the time) of 15 common non-pharmacologic CRF interventions derived by expert consensus, effectiveness of interventions, and barriers to care for CRF. The survey took approximately 7 min to complete and was distributed via emails or flyers to cancer support groups, listservs, recruitment flyers, and word of mouth. All analyses were descriptive, including frequencies and percentages, and were performed using Microsoft Excel.

**Results:**

One hundred ninety-seven rehabilitation providers completed the survey: physical therapy (60%), occupational therapy (20%), speech-language pathology (9%), and exercise physiology (5%). All disciplines indicated frequently applying more than one of the following within their CRF interventions: therapeutic activities (78%), aerobic/strengthening/flexibility exercise (73.1%), energy conservation strategies (57.4%), and self-management education (48.2%). All professions measured the effectiveness of their fatigue interventions using patients’ subjective reports (e.g., fatigue severity). Barriers to receiving care were “appointment fatigue” and financial constraints.

**Conclusions:**

When developing CRF clinical practice guidelines, current rehabilitation practice patterns and survivors’ barriers to care should be considered along with the highest level of evidence-based interventions.

**Supplementary Information:**

The online version contains supplementary material available at 10.1007/s00520-026-10717-8.

## Introduction

Cancer prevalence was estimated at 18.6 million persons in the United States in 2025; approximately 70% of those treated for cancer experience cancer-related fatigue (CRF) resulting in over 12 million people impacted by this condition [1–3]. Frequently, CRF negatively impacts function and quality of life (QOL), with studies reporting an inverse relationship between level of fatigue and functional abilities. CRF impacts individuals across the cancer care continuum [4, 5]. In those receiving active cancer treatment and in those living cancer-free, greater fatigue was associated with higher levels of self-reported disability, increased pain intensity, and lower levels of self-care efficacy [4–7]. Among individuals with a terminal cancer diagnosis receiving hospice care, fatigue was positively associated with depression and a strong predictor of diminished functional status [8]. Disability and function are important considerations for quality of life during and after cancer treatment as more than 70% of those with a history of cancer live at least five years after diagnosis [3].

Many studies have examined the efficacy and effectiveness of interventions to treat and manage CRF, including a number of systematic reviews summarizing the evidence [9–11]. Treatment options vary from pharmacological/medical (e.g., medication and blood support), to non-pharmacologic traditional conservative treatment (rehabilitation, exercise, and education) and complementary and alternative treatment (e.g., yoga, Tai Chi, and cognitive-behavioral therapy). While there is evidence supporting the application of many of these modalities in the treatment of CRF, there is little consensus on best practice from a non-pharmacologic standpoint. The European Society of Medical Oncology (ESMO), the National Comprehensive Cancer Network (NCCN), and the American Society of Clinical Oncology (ASCO) have published guidelines recommending rehabilitation services as a key component of a multimodal approach for the treatment of CRF. However, best practices for the rehabilitation of CRF have not yet been defined; this knowledge gap is a barrier to delivering the services that individuals with cancer need [12, 13].

The open-ended guideline recommendations and a variety of evidence leave healthcare providers’ without clear direction on best non-pharmacologic management practices for individuals with CRF. A qualitative study examined healthcare providers’ approach to managing CRF and found that insufficient knowledge, time for follow-up, accessible screening, and management tools or referral pathways were key barriers to providing evidence-based care [14]. The purposes of this study were to (a) evaluate current practice patterns for managing CRF and (b) describe barriers to implementing best practices. These findings can be used to inform rehabilitation clinical practice guideline development and implementation for CRF.

## Methods

### Study design

This was a cross-sectional study using an electronic survey that followed the STROBE and CHERRIES checklists [15, 16]. This study was approved by the University of South Carolina Institutional Review Board (Pro00127502) and received exempt status; therefore, consent was waived. Participants were neither incentivized nor reimbursed for their contributions. 

### Survey sample

To be eligible, survey respondents had to be 18 years of age or older and a healthcare rehabilitation provider within the United States (e.g., physical therapists (PT), occupational therapists (OT), speech-language pathologists (SLP), and exercise physiologists (EP)) who treats adults with cancer. Survey respondents were recruited via emails sent to available listservs (e.g., APTA Oncology [American Physical Therapy Association] and American Occupational Therapy Association) and by word of mouth over a 6-month period in 2023. Access to the survey was via QR codes or URL links to the survey and was open to anyone with the code or link. Respondents were not incentivized or remunerated for their contributions. The survey was available between March and November of 2023.

### Survey instrument

The survey was developed through an iterative process by the authors. Terms and concepts were operationally defined within each study question. Pilot testing was performed by asking nine rehabilitation providers (PT = 6, OT = 2, and EP = 1) who regularly treated adults with cancer to provide feedback about the survey. REDCap® version 12.5.9 (Vanderbilt University, Nashville, TN) electronic capture tools hosted at the University of South Carolina were used to host and distribute the survey. Branching logic was used to create questions in the surveys. If a respondent answered “no” to one question, the logic would take them to the next appropriate question. Modifications were made based on the nine rehabilitation providers’ suggestions, resulting in the final version. The survey was completed in approximately 5 min. Questions captured demographics, clinical background and experience, frequency of 15 multidisciplinary interventions for CRF (none of the time, some of the time, and all of the time), and single questions on which measures were used to measure CRF intervention effectiveness and what barriers to care were encountered. Participants were able to change answers/go back, if necessary, prior to submission of survey (see Supplementary information for questionnaire).

### Data analysis

All analyses were descriptive. Demographics are presented as means and standard deviations, and survey results are presented as numbers (frequencies/counts) and percentages. All percentages were calculated using the total number of respondents included.

## Results

The final sample included 198 respondents who completed the survey. Most healthcare-provider respondents were female (88%), white (90.4%), and younger than 44 years (63%). Healthcare rehabilitation professions represented included physical therapy (60%), occupational therapy (21%), speech-language pathology (14%), and exercise physiology (5%). Respondents had 11 or more years in their discipline (51%), had experience working with adults with cancer (29%), and had worked in an outpatient facility (70%). About 49% reported more than half of their caseload consisted of adults with cancer. Table 1 provides full details on the demographics of the respondents.

**Table 1:** Respondent demographics (*n* = 197)

Characteristic	*n*	%
Age group		
21–34 y	76	39
35–44 y	49	25
45–54 y	34	17
55–64 y	28	14
65 years or older	6	3
Prefer not to answer	4	2
Sex		
Female	174	88
Male	21	11
Prefer not to answer	2	1
Ethnicity		
Hispanic, Latino, or of Spanish origin	11	6
Not Hispanic, Latino, or of Spanish origin	186	94
Race		
White	176	89
Asian	11	6
Black or African American	3	1
Native Hawaiian or other Pacific Islander	1	0.5
Two or more races	5	3
Other	1	0.5
Country of residence		
United States	195	99
Other	2	1
Highest earned degree		
Bachelor of Science	34	17
Master of Science	42	21
Master of Arts	11	6
Clinical Doctorate	74	38
Doctorate (e.g., PhD, EdD, DHSc, and ScD)	27	14
Other	9	4
Primary discipline		
Exercise physiologist	10	5
Occupational therapist	52	26
Physical therapist	118	60
Speech and language pathologist	17	9
Experience in discipline		
Up to 1 y	8	4
1–3 y	21	11
4–10 y	68	35
11–20 y	44	22
21 y or longer	56	28
Experience with cancer patients		
Up to 1 y	17	9
1–3 y	47	24
4–10 y	75	38
11–20 y	33	17
21 y or longer	25	12
Clinical certifications relevant to cancer rehab		
Yes	117	59
No	80	41
Current practice setting		
Cancer center	52	
Inpatient rehabilitation	27	
Outpatient rehabilitation	135	
Academic institution	25	
Community fitness program	3	
Other	15	
Clinical caseload of adults with cancer		
10% or less	45	23
11–25%	29	15
26–50%	26	13
51–75%	28	14
76% or greater	69	35

### Types of interventions

**Fig. 1:**
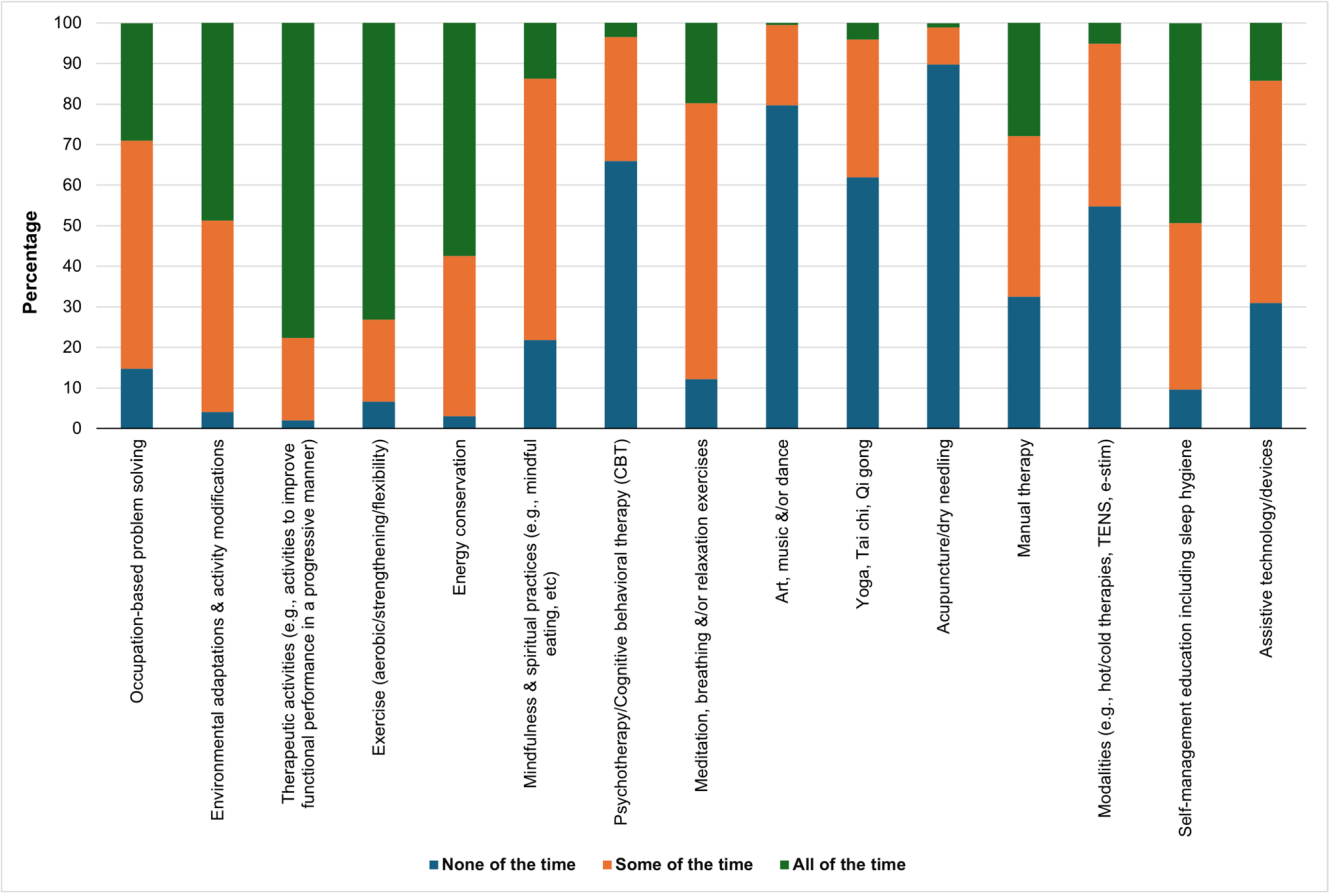
Frequency of interventions provided

All interventions included in the survey questionnaire (15 different areas) were used at varying levels by respondents. Respondents reported using the following CRF interventions “all of the time”: therapeutic activities (operationally defined as activities to improve functional performance in a progressive manner) [17] (78%), aerobic/strengthening/flexibility exercise (75%), energy conservation strategies (58%), and self-management education (50%). Complementary and alternative medicine strategies (e.g., meditation, dance, and Qi Gong) were the least reported interventions. When collapsing “all of the time” and “some of the time” categories, the most frequently used interventions included therapeutic activities (98%), energy conservation (97%), environmental adaptations and activity modifications (96%), exercise (93%), and self-management education (with sleep hygiene) (90%) (Fig. 1).

### Intervention effectiveness

Effectiveness of CRF interventions was frequently measured by patient subjective reports (89%), followed by subjective improvement in patient goals (80%) and patient-reported outcome measures (63%).

### Barriers to care

**Fig. 2:**
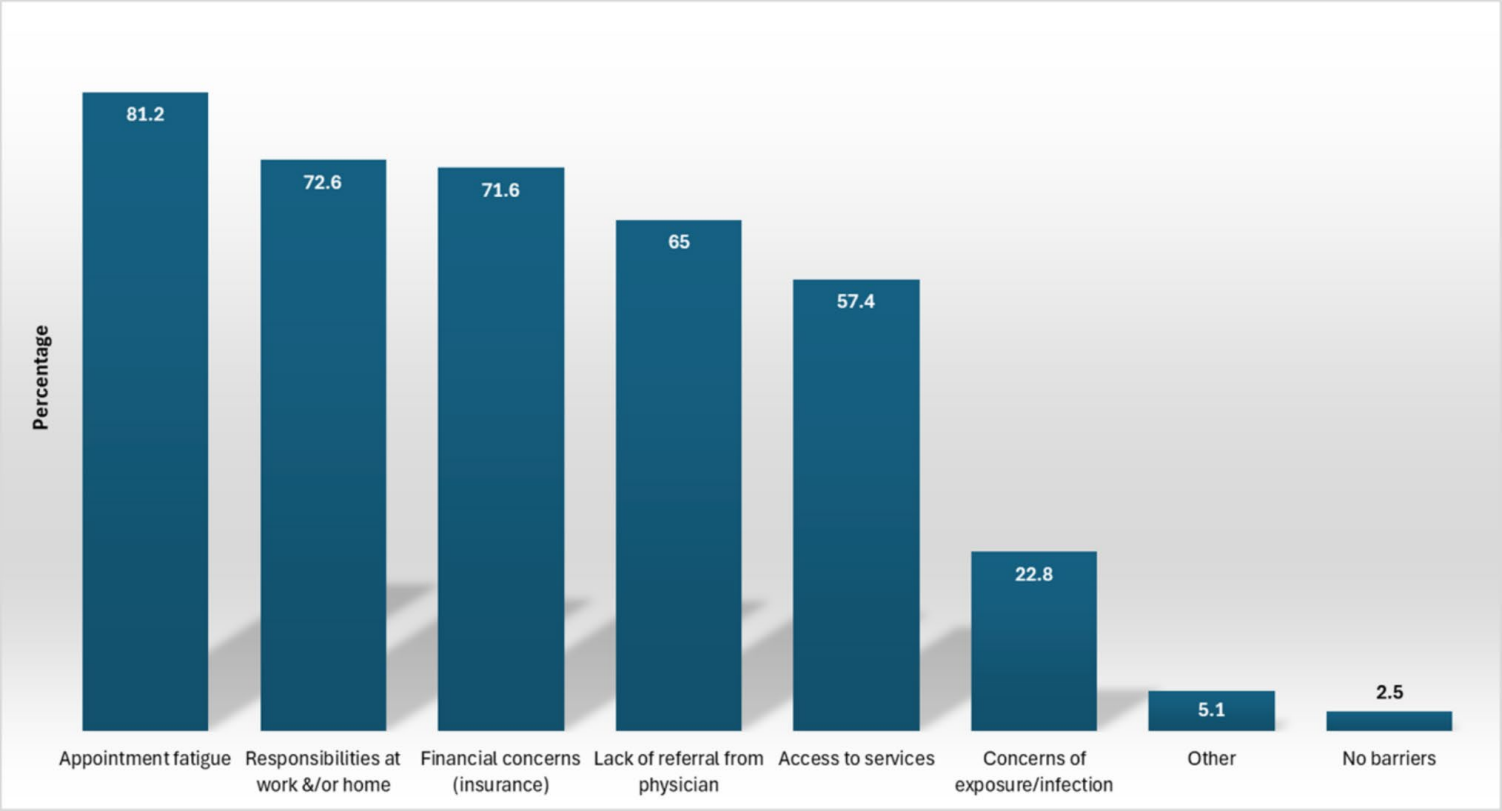
Barriers to providing care

The primary barriers to care were appointment fatigue (81%), financial concerns related to insurance (72%), and work/home responsibilities (73%). The ability to obtain care was also a barrier, including lack of referral from physicians (65%) and access to services (location or availability) (57%) (Fig. 2).

## Discussion

The purpose of this study was to evaluate current non-pharmacologic healthcare rehabilitation practice patterns for managing cancer-related fatigue with the goal of identifying barriers to implementation of best practices and directing the development of a clinical practice guideline for the management of CRF. Survey results highlight interesting trends in intervention strategies, how effectiveness is measured, and what barriers for care are present.

### Types of interventions

Over half of the rehabilitation providers who responded used more than one modality to treat CRF including therapeutic activities, exercise, energy conservation strategies, environmental and activity adaptation/modification, and self-management. While this survey did not collect details on which therapeutic activities were employed, the authors defined this as activities to improve functional performance in a progressive manner. Little research is available to substantiate the effectiveness of therapeutic activity in the management of CRF, or even within a general population. Most research centers on the effectiveness of therapeutic exercise rather than activity aimed at functional recovery. Improving function reflects a patient-centered goal compared to improving strength and endurance which are means to restore function. Additionally, therapeutic activities may be a billing construct related to interventions aimed at improving function and therefore not directly searchable in the literature [18]. Of the healthcare providers who responded “some” or “all of the time” in using therapeutic activities, 80% were those who bill this way (PT/OT/SLP). This is an area that needs further investigation; are healthcare providers using therapeutic activity to reach functional goals or is it a specific intervention? Exercise is recommended by the American College of Sports Medicine (ACSM) for individuals living with a cancer diagnosis. Multiple papers support the impact of exercise in cancer prevention and risk reduction [19–22], but more importantly, strong evidence for the mitigation of CRF through exercise is highlighted in the ACSM Exercise is Medicine program [19–22]. Studies support the implementation of moderate-intensity aerobic exercise combined with resistance exercise performed 2–3 times per week to significantly reduce fatigue levels [19]. Furthermore, moderate to vigorous-intensity exercise had the greatest effect on reducing CRF. Evidence for dosing was less clear, with suggestions that the program must be a minimum of 12 weeks and each session a minimum of 30 min [19].

Energy conservation strategies, including activity planning and pacing, prioritization of activity, and modification of performance of activity, are commonly used in chronic conditions to mitigate the impact of taxing activities on energy levels. Emerging evidence supports the use of energy conservation strategies in CRF management. A randomized controlled trial (*n* = 135) focused on energy conservation strategies combined with health promotion reported that the intervention group demonstrated greater reductions in fatigue compared to a control group in a population of women with breast cancer [23]. Our findings show that energy conservation strategies are employed nearly all the time to manage CRF, yet a consensus on recommending energy conservation is lacking in existing practice guidelines [12, 13]. More research is needed to validate energy conservation use as an effective treatment for CRF. Practice patterns reported by the respondents in this study appear to correlate with the literature in implementing a multimodal approach to CRF management. However, what is still lacking is a clear approach to determining who will benefit from which interventions, and what dosage is appropriate. A clinical practice guideline providing greater specificity is needed to improve clinical care for those with CRF.

The importance of empowering those with CRF to manage their condition independently is a commonly cited goal in CRF treatment. Since CRF affects most individuals diagnosed with cancer, determining the best self-management strategies is critical. A recent systematic review including 7383 individuals enrolled in 50 different studies found self-management delivered by healthcare providers is effective in reducing fatigue [24]. This suggests rehabilitation professionals play an important role in providing individuals with the skills to manage their fatigue.

eHealth (use of synchronous or asynchronous computer-based intervention) and mHealth (use of mobile devices for intervention) are growing in popularity, and several studies have examined the use of an application specifically designed to provide individuals with cancer-related fatigue some tools to manage their fatigue independently. The Untire™ app was found to reduce fatigue catastrophizing and depression while increasing mindfulness, with the impact of reducing fatigue severity and interference [25, 26]. Rehabilitation professionals have multiple treatment options and modes of delivery to provide CRF self-care management skills.

### Effectiveness of treatment

Rehabilitation professionals in this study noted multiple methods to assess the effectiveness of CRF interventions; many methods incorporated some type of self-report by the individual, including subjective report of fatigue level, goal attainment, and other patient-reported outcome measures. A previous CPG on the screening and assessment of CRF highlighted recommended tools for these purposes. However, in researching the tools for CRF determination, many lack psychometric information on sensitivity to change [27]. Without psychometric information on sensitivity to change, these tools should be used with caution when measuring outcomes or effectiveness of interventions. This leaves simple patient report as the sole way to measure effectiveness of treatment. Future measurement studies should explore the use of objective measures and evaluate current subjective measures’ sensitivity to change to establish meaningful ways to measure the effectiveness of these interventions.

### Barriers

Treatment barriers are important to understand in order to address the needs of those with CRF. Treating cancer includes a large multidisciplinary team; this may result in having multiple appointments at different times and places. Individuals with cancer frequently report that scheduling, coordinating, and attending these appointments can be fatiguing, leading to reluctance in attending additional appointments to address CRF [28]. Centralized comprehensive cancer centers can reduce extra traveling and the need for coordinating appointments, thereby reducing cognitive and physical fatigue.

Financial concerns and work/home responsibilities are significant barriers to care and were cited equally by respondents. Little research describing their association to barriers to CRF management is available; however, an analysis of multinational healthcare (including the United States) expenditures revealed that those with lower incomes had a greater incidence of foregone care [29]. However, while lack of physician or oncology team referral, either from lack of awareness or lack of access, was not the greatest barrier identified in our study, a significant body of evidence supports that this is a real barrier for those with CRF [28, 30, 31]. Individuals are typically unsure of whom they should discuss their fatigue with, have a fear of a negative perception if they complain [30] and are concerned they would distract the physician from treating their disease [31]. Understanding barriers is essential to optimizing care for those with CRF.

### Study limitations

The return rate was not calculated due to a broad recruitment strategy; however, this survey had a robust response rate, and the questions were evaluated by expert consensus prior to deployment, which provides rich data for understanding the provision of care for those with CRF. However, there are some structural aspects of the study that limit comprehensive analysis. Some terms, while operationally defined, lacked rich definitions and could be interpreted differently by respondents. A lack of detailed specificity for the types of treatments—including therapeutic activities, exercise, and environmental adaptation—limits a detailed understanding of types of treatments. We also did not ask questions about dosing—how often and how long individuals were treated. We did not include nutritional strategies (either supplements or dietary) as the author team focused on specific rehabilitative professionals and interventions within their scope of practice. Lastly, we had a greater number of physical therapists than other health professionals answering our survey, potentially skewing treatment frequencies to those typically rendered by physical therapists.

### Future directions

The results of this study do offer suggestions for further research—including detailed descriptions of (a) the types of CRF treatments and how they are delivered, (b) who delivers each CRF treatment type, and (c) modes of delivery and settings (hospital, community etc.). These descriptions can further improve the understanding of care for those with CRF to improve access and outcomes.

## Conclusions

The findings of this study provide a greater understanding of the current practice patterns to manage CRF. These practice patterns do not consistently reflect evidence for effective treatment of CRF. Understanding barriers to implementing evidence-based care is important in developing clinical practice guidelines that not only recommend interventions based on scientific evidence but also consider real-world clinical practice.

## Supplementary Information

Below is the link to the electronic supplementary material.

Supplementary file1 (PDF 51.4 KB)

## Data Availability

Data can be provided at the request of individuals.
