# Supplementary material for: Current non-pharmacological practices for cancer-related fatigue in oncology rehabilitation: results of a national provider survey
Source: Support Care Cancer. 2026 May 8;34(6):517. doi: 10.1007/s00520-026-10717-8 (PMC13152946; doi:10.1007/s00520-026-10717-8)
Supplement: Supplementary file 1 — Supplementary file1 (PDF 51.4 KB) [file 520_2026_10717_MOESM1_ESM.pdf]

# Rehab provider CRF intervention survey

Please complete the survey below.

Thank you!

| | |
|---|---|
| What age group categorizes you? | ◯ 21- 34 years<br>◯ 35 - 44 years<br>◯ 45 - 54 years<br>◯ 55 - 64 years<br>◯ 65 and over<br>◯ Prefer not to answer |
| What sex do you identify with? | ◯ Female<br>◯ Male<br>◯ Prefer not to answer |
| Are you Hispanic, Latino or of Spanish origin? | ◯ Yes<br>◯ No |
| How would you describe yourself? | ◯ American Indian or Alaska Native<br>◯ Asian<br>◯ Black or African American<br>◯ Native Hawaiian or other Pacific Islander<br>◯ White<br>◯ Two or more Races<br>◯ Other |
| If selected "other", please describe: | __________________________________ |
| What country do you currently reside in? | ◯ United States<br>◯ Other |
| If "other" country, please describe: | __________________________________ |
| What is your highest earned degree? | ◯ Bachelor of Science<br>◯ Master of Science<br>◯ Master of Arts<br>◯ Clinical Doctorate<br>◯ Doctorate (e.g., PhD, EdD, DHSc, ScD)<br>◯ Other |
| If "other" degree, please describe: | __________________________________ |
| What is your primary discipline (e.g., in which you are addressing cancer-related fatigue)? | ◯ Exercise Science<br>◯ Physical Therapy<br>◯ Occupational Therapy<br>◯ Speech and Language Pathology<br>◯ Other |
| If "other" discipline, please describe: | __________________________________ |

| | |
|---|---|
| How many total years of experience do you have in your discipline? (e,g, I've been an OT for 10 years) | ◯ < 1 year<br>◯ 1-3 years<br>◯ 4-10 years<br>◯ 11-20 years<br>◯ over 21 years |
| How many total years of experience do you have working with individuals who have or had a cancer diagnosis? | ◯ < 1 year<br>◯ 1-3 years<br>◯ 4-10 years<br>◯ 11-20 years<br>◯ over 21 years |
| Do you have any clinical certifications relevant to exercise oncology and/or cancer rehabilitation? (e.g., Cancer exercise trainer, Board-certification in Oncologic Physical Therapy, Lymphedema specialist, etc.) | ◯ Yes<br>◯ No |
| If "yes", please describe: | __________________________ |
| What is your current practice setting? (select all that apply) | ☐ Cancer center<br>☐ Inpatient rehabilitation<br>☐ Outpatient rehabilitation<br>☐ Academic institution<br>☐ Community fitness program<br>☐ Other |
| If "other" practice setting, please describe: | __________________________ |
| On average, what portion of your clinical case load is adults living with and beyond cancer? | ◯ 10% or less<br>◯ 11 - 25%<br>◯ 26 - 50%<br>◯ 51 - 75%<br>◯ 76% or greater |

**Below is a list of multidisciplinary interventions. Many of these may not apply to you or your discipline.**

**Indicate how frequently you use the following interventions when managing cancer-related fatigue**

| | None of the time | Some of the time | All of the time |
|---|---|---|---|
| Occupation-based problem solving | ◯ | ◯ | ◯ |
| Environmental adaptations and activity modifications | ◯ | ◯ | ◯ |

| | | | |
|---|---|---|---|
| Therapeutic activities (e.g., activities to improve functional performance in a progressive manner) | ◯ | ◯ | ◯ |
| Exercise (aerobic/strengthening/flexibility) | ◯ | ◯ | ◯ |
| Energy conservation | ◯ | ◯ | ◯ |
| Mindfulness & spiritual practices (e.g., mindful eating, etc) | ◯ | ◯ | ◯ |
| Psychotherapy/Cognitive behavioral therapy (CBT) | ◯ | ◯ | ◯ |
| Meditation, breathing &/or relaxation exercises | ◯ | ◯ | ◯ |
| Art, music &/or dance | ◯ | ◯ | ◯ |
| Yoga, Tai chi, Qi gong | ◯ | ◯ | ◯ |
| Acupuncture/dry needling | ◯ | ◯ | ◯ |
| Manual therapy | ◯ | ◯ | ◯ |
| Modalities (e.g., hot/cold therapies, TENS, e-stim) | ◯ | ◯ | ◯ |
| Self-management education including sleep hygiene | ◯ | ◯ | ◯ |
| Assistive technology/devices | ◯ | ◯ | ◯ |

---

Do you perform any additional interventions not listed above?

◯ Yes
◯ No

---

Please list these other interventions:

__________________________________

---

What percentage of indivdiuals with cancer-related fatigue did you apply one of the interventions listed above?

◯ 10% or less
◯ 11 - 25%
◯ 26 - 50%
◯ 51 - 75%
◯ 76% and greater

---

How do you measure the effectiveness of the interventions for cancer-related fatigue? (select all that apply)

☐ Patient subjective report (specifically related to fatigue)
☐ Subjective report of improvement in patient-centered goals
☐ Patient-reported outcome measure(s) (e.g., Brief Fatigue Inventory, Piper Fatigue Scale, One-item Fatigue Scale, etc.)
☐ Improvement in other medical outcomes
☐ No outcomes used
☐ Other

---

If selected "improvement in other medical outcomes", please describe these:

__________________________________

---

If "other", please describe:

__________________________________

| | |
|---|---|
| Indicate barriers to care for individuals with cancer-related fatigue? (select all that apply) | ☐ Access to services<br>☐ Financial concerns (e.g., insurance coverage, copays, etc.)<br>☐ Concerns regarding exposure/infection<br>☐ Fatigue (e.g., appointment fatigue)<br>☐ Patient conflicts (e.g., social responsibilities at work and/or home)<br>☐ Lack of referral from physician and/or oncology team<br>☐ No barriers<br>☐ Other |
| If "other" barrier, please describe: | __________________________________ |
